# Pattern Formation in Catalytic H_2_ Oxidation on Rh: Zooming in by Correlative Microscopy

**DOI:** 10.1021/acscatal.2c03692

**Published:** 2022-09-19

**Authors:** Johannes Zeininger, Philipp Winkler, Maximilian Raab, Yuri Suchorski, Mauricio J. Prieto, Liviu C. Tănase, Lucas de Souza Caldas, Aarti Tiwari, Thomas Schmidt, Michael Stöger-Pollach, Andreas Steiger-Thirsfeld, Beatriz Roldan Cuenya, Günther Rupprechter

**Affiliations:** † Institute of Materials Chemistry, TU Wien, Getreidemarkt 9, 1060 Vienna, Austria; ‡ Department of Interface Science, Fritz-Haber-Institut der Max-Planck Gesellschaft, Faradayweg 4-6, D-14195 Berlin, Germany; § University Service Center for Transmission Electron Microscopy, TU Wien, Wiedner Hauptstraße 8-10, 1040 Vienna, Austria

**Keywords:** catalytic hydrogen oxidation, correlative microscopy, photoemission electron microscopy, low-energy electron microscopy, microkinetic modeling, Monte Carlo modeling

## Abstract

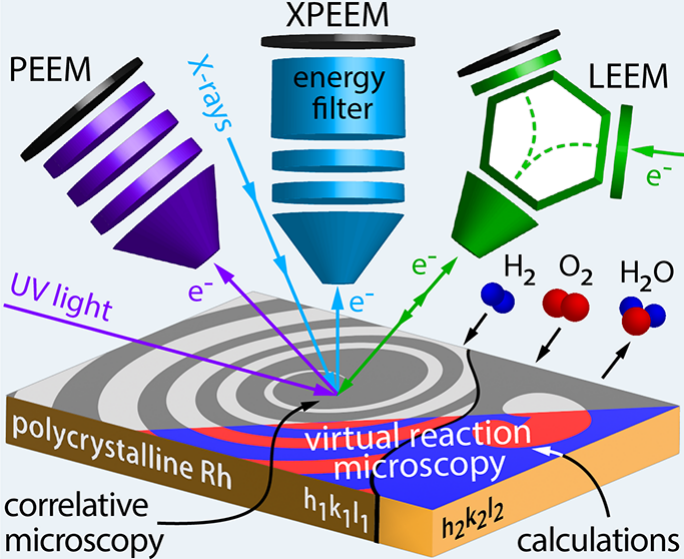

Spatio-temporal nonuniformities in H_2_ oxidation on individual Rh(*h* *k* *l*) domains of a polycrystalline Rh foil were studied in the 10^–6^ mbar pressure range by photoemission electron microscopy (PEEM), X-ray photoemission electron microscopy (XPEEM), and low-energy electron microscopy (LEEM). The latter two were used for in situ correlative microscopy to zoom in with significantly higher lateral resolution, allowing detection of an unusual island-mediated oxygen front propagation during kinetic transitions. The origin of the island-mediated front propagation was rationalized by model calculations based on a hybrid approach of microkinetic modeling and Monte Carlo simulations.

## Introduction

Heterogeneous catalysis is one of the key enablers of sustainable energy generation and storage. For example, renewable energy can be stored in the chemical bonds of hydrogen and released on-demand via H_2_ oxidation in a fuel cell,^1^ rendering a fundamental understanding crucial for efficient operation.^2^

The interaction between active sites in a heterogeneous catalytic reaction may lead to spatial nonuniformities of the local reaction rates, which may also vary in time.^3−5^ Such pattern formation has often been observed for reactions with oscillatory or multistable kinetics, such as H_2_ or CO oxidation, NO reduction, or NO_2_ reduction on noble metals,^5−9^ and has been a subject of longstanding studies and several reviews.^10−12^ Recently, single-crystal studies were extended to more realistic systems, and a number of effects were detected that influence the spatio-temporal structures: multifrequential oscillations, both in μm-^13^ and nm-sized^14^ systems, frequency transformation by grain boundaries^15^ and by atomic rows,^14^ coexisting multi-states^16^ and nm scale reaction pacemakers.^17^ These previous studies have demonstrated the plethora of novel effects that may occur in surface reactions. However, studying aspects such as communication between adjacent domains or facets acting as individual oscillators still remains a challenge. The same concerns the mechanisms of pattern formation on a heterogeneous surface with varying sample topology. To address these challenges, it is necessary to significantly improve the lateral resolution, preferentially combined with chemical sensitivity. Still, conventional modeling approaches have typically been focused on either mean-field kinetic modeling well applicable to extended homogeneous systems^13,15,16^ or Monte Carlo simulations on the traditional atomic length and time scales.^11,18^ To combine both approaches for the simulation of processes on mesoscopic heterogeneous surfaces is another challenge in studying surface reactions.

To reveal the mechanisms of spatio-temporal effects, it is advantageous to study these dynamic processes in situ at different length scales using different microscopy techniques. Such an approach was recently applied to monitor CO and H_2_ oxidation at different length scales on rhodium samples with different morphologies, i.e., single crystals, μm-sized domains of a polycrystalline foil, and facets of a nanotip.^19,20^

The most straightforward way to draw correct conclusions from such studies is to apply different microscopies to the same surface structure, if possible, under the same conditions. The approach stems from biological research, where the first efforts of correlative light and electron microscopy (CLEM) on the same cell/tissue structures were made already in the 1970s.^21,22^ In the meantime, CLEM has been extended to a broad category of methods combining any type of light and electron microscopy on the same sample.^23^ Actually, other imaging techniques such as atomic force microscopy (AFM), X-ray tomography, and scanning electron microscopy (SEM) are also successfully used in a correlative microscopy approach.^24,25^ In the last few decades, correlative analysis has made a vast impact in materials research, combining, e.g., transmission electron microscopy (TEM) with atom probe tomography (APT)^26^ or Raman with SEM.^27,28^ Using protective coatings, changes in the sample resulting from transfer (air exposure) between different devices can be avoided.^29^ In catalysis, the correlative microscopy approach, often applied in different setups,^30−32^ has reached its highest development stage when different microscopies, e.g., single-molecule fluorescence microscopy (SMF) and TEM are combined in one instrument.^33^ Dual microscopy combinations, such as LEEM/PEEM,^34^ LEEM/XPEEM,^35^ or PEEM/SPEM,^36^ were also previously used to study potassium redistribution in alkali-promoted H_2_ oxidation on Rh(110).

Here, we present a refinement of this concept, combining three different microscopic techniques (PEEM, XPEEM, and LEEM) for in situ imaging of catalytic H_2_ oxidation on the same structures of the same Rh sample, i.e., in a correlative approach. Two of them, XPEEM (with chemical sensitivity) and LEEM (with a resolution of 2.6 nm, i.e., much better than in conventional PEEM), were even combined in a single setup and thus under truly identical reaction conditions. Additionally, the selected small area (1.5 μm diameter) electron diffraction (μ-LEED) mode of LEEM was used for the determination of the local crystallography.

As a model system, individual differently oriented well-defined μm-sized Rh(*h k l*) domains of a polycrystalline Rh foil were used, all being automatically under identical reaction conditions. The ongoing catalytic reaction was monitored in situ by all three microscopies, and an analysis of the recorded video frames provides time-resolved data of the observed process (kinetics by imaging^37^).

**Figure 1:**
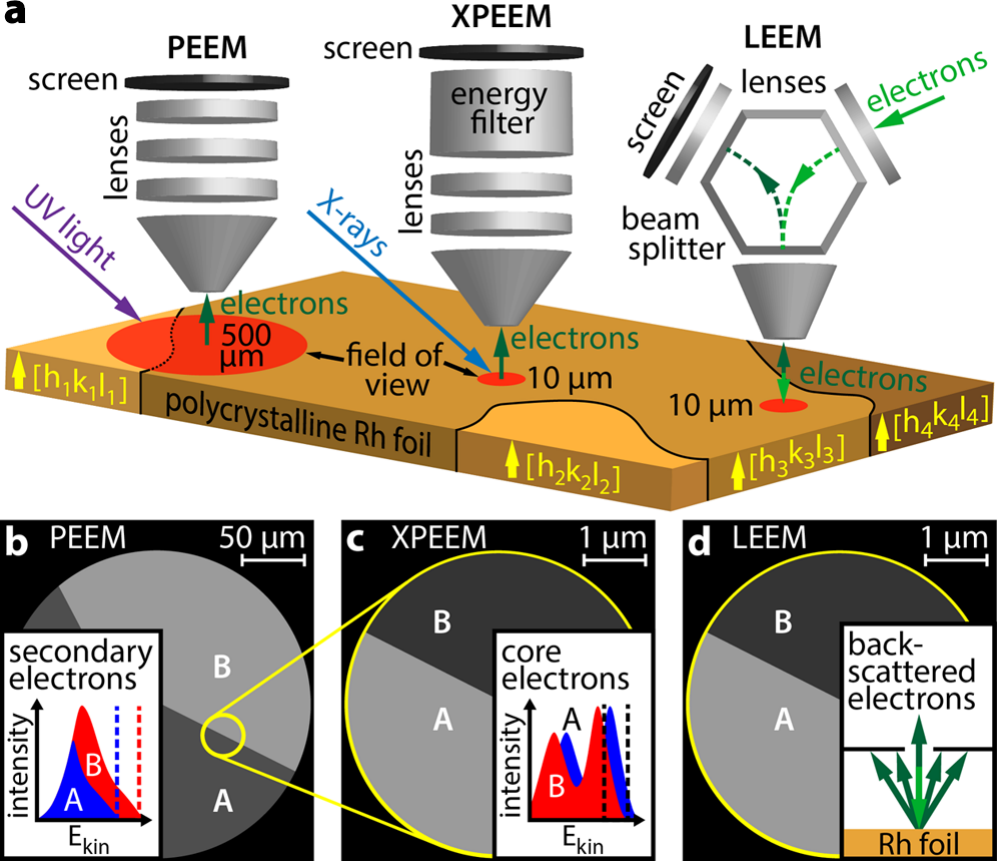
Correlative microscopy approach: (a) different information is collected in situ from the same individual domain of a polycrystalline Rh foil using different imaging techniques; (b) in PEEM with UV-light excitation, inelastically scattered photoemitted electrons create the image; (c) in XPEEM with X-ray excitation, energy-filtered core level electrons provide the image contrast; and (d) in LEEM, elastically backscattered electrons are utilized. The bright-field imaging mode with a contrast aperture selecting the specularly reflected electron beam was used.

The approach is illustrated in Figure 1a: an individual Rh(*h k l*) domain can be selected from the surface structure library (XPEEM, LEEM), or even a few different domains in the field of view can be studied simultaneously (PEEM). The different employed microscopy techniques vary in magnification, resolution, and origin of the information carriers: secondary (inelastically scattered) electrons form the image in PEEM with UV-light excitation (Figure 1b), core level electrons in XPEEM (Figure 1c), and backscattered low-energy electrons in LEEM (bright-field imaging mode was used, Figure 1d). All available Rh(*h k l*) domains were crystallographically characterized beforehand by electron backscatter diffraction (EBSD). No electron beam and X-ray beam effects were observed in the present experiments. This was directly proven by in situ switching between the XPEEM and LEEM modes in the present study and between LEEM and PEEM^34^ as well as between PEEM and metastable impact electron emission microscopy (MIEEM), known as the least invasive surface analysis method,^38^ in earlier experiments on H_2_ oxidation on Rh.

In the present work, we have taken up the abovementioned pattern formation challenge by studying catalytic H_2_ oxidation on Rh, an often-studied reaction that still keeps the mechanisms of spatial propagation hidden. Due to the well-known reaction mechanism, the reaction is well suited for the application of novel modeling approaches. A combined application of PEEM, LEEM, with its significantly higher resolution, and XPEEM, with its chemical sensitivity, all used in situ on the same surface regions, has allowed us to gain unprecedented insights into the mechanisms behind the formation of spatio-temporal patterns. A novel hybrid modeling approach combining microkinetic modeling and Monte Carlo simulations corroborated the experimental data well and allowed time-dependent simulations in a kind of virtual reaction microscopy.

## Results and Discussion

### PEEM Studies

The ongoing H_2_ oxidation reaction on Rh was visualized in an ultrahigh vacuum (UHV) PEEM setup operated as a flow reactor. The setup is equipped with a deuterium discharge lamp (Heraeus D200F, photon energy ∼6.5 eV) for UV illumination, a Staib Instruments PEEM 150 system, a high-speed CCD camera (Hamamatsu C11440-42U30), a quadrupole mass spectrometer (MKS e-Vison 2), gas dosing (Ar, H_2_, O_2_; purity 99.999%), and sample cleaning facilities. More details on the experimental procedures are given in the Supporting Information.

**Figure 2:**
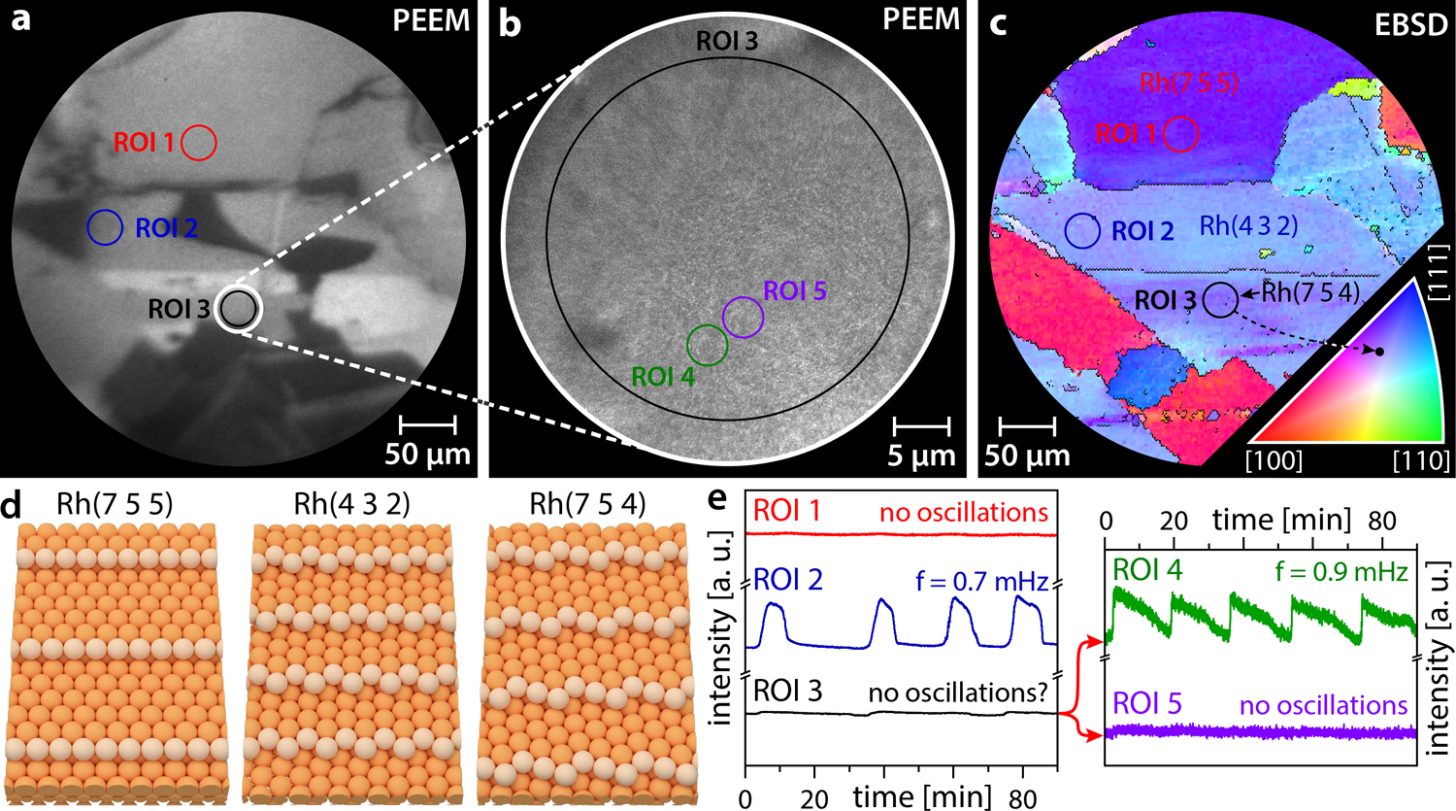
PEEM zoom-in on catalytic H_2_ oxidation on a polycrystalline Rh foil at *p*H_2_ = 4.5 × 10^–7^ mbar, *p*O_2_ = 5.0 × 10^–7^ mbar, and *T* = 468 K: (a) PEEM snapshot taken during the ongoing reaction with three regions of interest (ROIs) marked; (b) electron-optical zoom-in (10-fold higher magnification) PEEM snapshot of the region marked by a white circle in panel (a); (c) EBSD map and crystallographic orientations (Miller indices) of the same region with the same ROIs as in panel (a); the legend for the color code is given as an inset in the lower right corner; (d) ball models of the Rh(7 5 5), Rh(4 3 2), and Rh(7 5 4) surfaces; and (e) time series of the local PEEM intensities obtained for the structurally different ROIs marked in panels (a) and (b).

Figure 2a shows a PEEM snapshot from a video sequence recorded during the ongoing reaction at constant *T* = 468 K, *p*H_2_ = 4.5 × 10^–7^ mbar, *p*O_2_ = 5.0 × 10^–7^ mbar. The image contrast results from the differences in the local work function, which in turn depends on the adsorbate coverage: the catalytically inactive (oxygen-covered) Rh(*h k l*) surface appears generally darker than the catalytically active (low H_ads_ and O_ads_ coverage) surface due to the higher work function of the oxygen-covered Rh surface. An illustration of the catalytically active and inactive states is given in the Supporting Information (Figure S2).

To study the reaction behavior on structurally different Rh(*h k l*) domains, the local PEEM intensity was analyzed for different regions of interest (ROIs) on the surface. Three ROIs were placed on different Rh(*h k l*) regions, crystallographically defined by EBSD in a field emission scanning electron microscope (FEI Quanta 200F, further details are given in the Supporting Information). The corresponding local PEEM intensity time series are shown in Figure 2e, revealing cardinal differences in the kinetic behavior of different regions: ROI 1 constantly remains in the catalytically active state, whereas ROI 2 shows a self-sustaining oscillating mode of the reaction (frequency 0.7 mHz). The reaction course for ROI 3 is actually not clear: oscillatory behavior can neither be presumed nor excluded.

To explain the remarkable differences in the local reaction behavior, we refer to the color-coded EBSD map in Figure 2c, with the color code given as an inset in the lower right corner. The EBSD characterization reveals that ROIs 1 and 2 are located on homogeneous Rh(7 5 5) and Rh(4 3 2) domains, respectively, while ROI 3 was placed within a domain of a rather heterogeneous surface structure.

The reaction modes observed at the present conditions for the homogeneous Rh(7 5 5) and Rh(4 3 2) domains can be understood in view of recent studies in which different reaction modes of H_2_ oxidation were simultaneously observed on adjacent crystallographically different regions.^16^ The Rh(7 5 5) surface (ROI 1) is characterized by [1 1 1]-type terraces in combination with nonkinked [1 0 0]-type step edges. The Rh(4 3 2) surface (ROI 2) is also characterized by [1 1 1]-type terraces but combined with kinked [2 1 0]-type step edges. This is illustrated in atomic ball models in Figure 2d. The structural differences lead to differing kinetic behavior: as recently shown, the formation and depletion of subsurface oxygen on the Rh(*h k l*) surfaces and the resulting enabling or inhibition of dissociative hydrogen adsorption may lead to self-sustaining oscillations in H_2_ oxidation, with the activation energy of the oxygen incorporation governing the oscillation frequency.^13−16^ An illustration of the oscillation cycle and further details of the mechanism of the oscillations are presented in the Supporting Information (Figure S2) and have been extensively discussed previously.^13−16^ For Rh, the activation energy of subsurface oxygen formation strongly varies with the surface roughness, with the kink sites playing a particular role.^39^ The Rh(4 3 2) surface exhibits kinked step edges, which accelerates the formation/depletion of subsurface oxygen compared to the nonkinked Rh(7 5 5) surface. This leads to the observed oscillatory reaction behavior on the Rh(4 3 2) surface, whereas the Rh(7 5 5) surface remains in the catalytically active state at the present conditions.

To evaluate the reaction behavior within ROI 3, where the discrimination between a possible steady state and oscillatory behavior seems difficult, the reaction was monitored at the same conditions but with an electron-optical magnification 10 times higher than that of Figure 2a. Figure 2b shows the corresponding PEEM video frame, revealing instable bright and dark spots that are invisible at lower magnification. Placing additional ROIs (ROIs 4 and 5) on such spots and evaluating the local PEEM intensities yield the two time series displayed in the right panel of Figure 2e. While the PEEM intensity read out from ROI 5 remains constant, the intensity within ROI 4 oscillates with a frequency of 0.9 mHz. Apparently, on a structurally heterogeneous Rh surface, similar phenomena as represented by the three curves in the left panel of Figure 2e take place but on a much smaller length scale, possibly due to a significantly smaller correlation length of individual oscillating regions due to structural peculiarities. The black curve (ROI 3) in Figure 2e thus represents a weighted sum of both oscillating and nonoscillating surface regions, which accounts for the difficulties in interpreting the behavior when averaging over the whole ROI. To understand these phenomena, alternative imaging methods with much higher magnification, resolution, and chemical contrast appear promising.

### Correlative LEEM and XPEEM Studies

As such alternative imaging methods, the LEEM and XPEEM modes of the aberration-corrected photoemission electron microscope equipped with an imaging energy analyzer (SMART) at the UE49-PGM beamline of the BESSY II synchrotron light source in Berlin were used (resolution limits of 2.6 and 18 nm for LEEM and XPEEM, respectively).^40,41^ These modes of the SMART microscope, described in detail elsewhere,^42,43^ were applied in a correlative microscopy approach, combining the imaging of the same sample by backscattered low-energy electrons (LEEM) and X-ray photoemitted electrons (XPEEM). A 7.5 μm field of view was chosen for the present studies to capture the processes of interest. The local surface crystallography was determined by μ-LEED (see the Supporting Information), while μ-XPS was used for local chemical analysis. The UHV chamber of the SMART microscope was used as a flow reactor for in situ visualization of catalytic H_2_ oxidation on the same sample as in the PEEM studies. The bright-field LEEM mode using the (00)-diffracted electron beam for imaging was applied to monitor the reaction. The LEEM image contains structural contrast but no direct chemical information, which can be gained by XPEEM, however.

**Figure 3:**
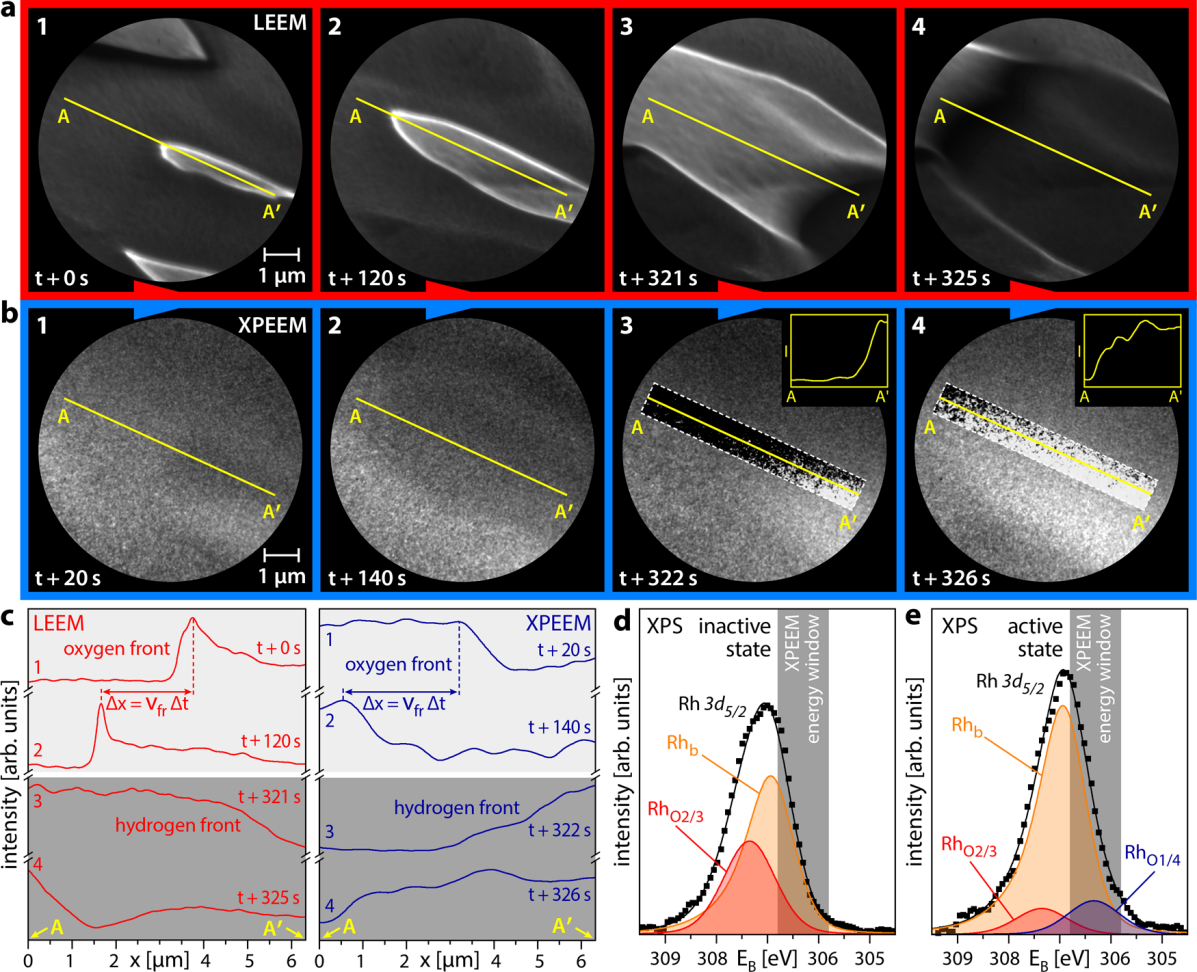
Reaction front identification using correlative microscopy: (a) bright-field LEEM images of oxygen front propagation (frames 1 and 2) and hydrogen front propagation (frames 3 and 4) on the Rh(7 5 4) domain at *p*H_2_ = 4.5 × 10^–7^ mbar, *p*O_2_ = 5.0 × 10^–7^ mbar, *T* = 468 K, and electron energy = 2.5 eV; (b) the same, but imaged with XPEEM, the used binding energy window is shaded gray in panels (d) and (e). In the dashed rectangular regions, the image contrast is enhanced locally for better front visibility, the insets in frames 3 and 4 illustrate the front positions; (c) local LEEM (left) and XPEEM (right) image intensity profiles obtained from the original data along the A–A′ line in panels (a) and (b), the principle of the determination of the front propagation velocity is illustrated; (d) Rh 3d XPS spectra locally measured on the Rh(7 5 4) domain for the catalytically inactive state in H_2_ oxidation. Deconvolution of the peaks provides the Rh_b_ and Rh_O_2/3__ components; (e) the same, but for the catalytically active state, with an additional Rh_O_1/4__ component present in the deconvolution, identifying the hydrogen front.

Figure 3a shows selected LEEM video frames of the reaction front propagation during self-sustained oscillations on a Rh(7 5 4) domain at constant *T* = 468 K, *p*H_2_ = 4.5 × 10^–7^ mbar, *p*O_2_ = 5.0 × 10^–7^ mbar. Two different types of reaction fronts were observed: a slow-moving front in frames 1 and 2 (time difference of 120 s) and a fast-moving front in frames 3 and 4 (time difference of 4 s). To understand the chemical information encoded in the image contrast in Figure 3a, similar series of Rh 3d XPEEM video frames (hν = 433 eV) were acquired, as exemplarily shown in Figure 3b. The image contrast in Figure 3b results from an energy window for Rh 3d XPEEM imaging being chosen in the range 305.8–306.8 eV, marked in gray in Figure 3d,e. The choice of this energy window is based on the local Rh 3d μ-XPS spectra of the catalytically inactive and active states on the Rh(7 5 4) domain, shown in Figure 3d,e, respectively. Deconvolution of the measured XPS spectra was performed based on literature data and is discussed in refs (16, 44−47). The spectra include a Rh bulk component (Rh_b_) and two components for two different adsorbed oxygen species (Rh_O_2/3__ and Rh_O_1/4__, where *i* and *j* in Rh_O_*i*/*j*__ denote the number of O atoms each Rh surface atom is bound to and the number of Rh surface atoms each O atom is bound to, respectively).

The presence of the Rh_O_1/4__ component is unambiguously related to the active state in catalytic H_2_ oxidation. The physical reasons for this relation are clarified in our previous XPS study.^16^ By choosing the proper energy window, this component can thus be used as a basis for the contrast in XPEEM, imaging the extent of the active state on the surface. For better visualization of the reaction fronts, the image contrast of the original XPEEM video frames was locally enhanced within the white dashed rectangles in frames 3 and 4 of Figure 3b. Line profiles along the A–A′ line, shown in the insets of frames 3 and 4 of Figure 3b, illustrate the determination of reaction front positions. Analysis of the front positions provides the velocities of the reaction fronts, as shown in Figure 3c.

Comparison of the front velocities in the video files then allows relating the image contrast in LEEM and XPEEM: at the used electron energies of 2.5–3.5 eV, the inactive state (high oxygen coverage) appears bright in LEEM but dark in XPEEM, whereas the active state (low hydrogen and oxygen coverage) is dark in LEEM but bright in XPEEM. This means that the transition from dark to bright in LEEM equals a transition from bright to dark in XPEEM and vice versa.

**Figure 4:**
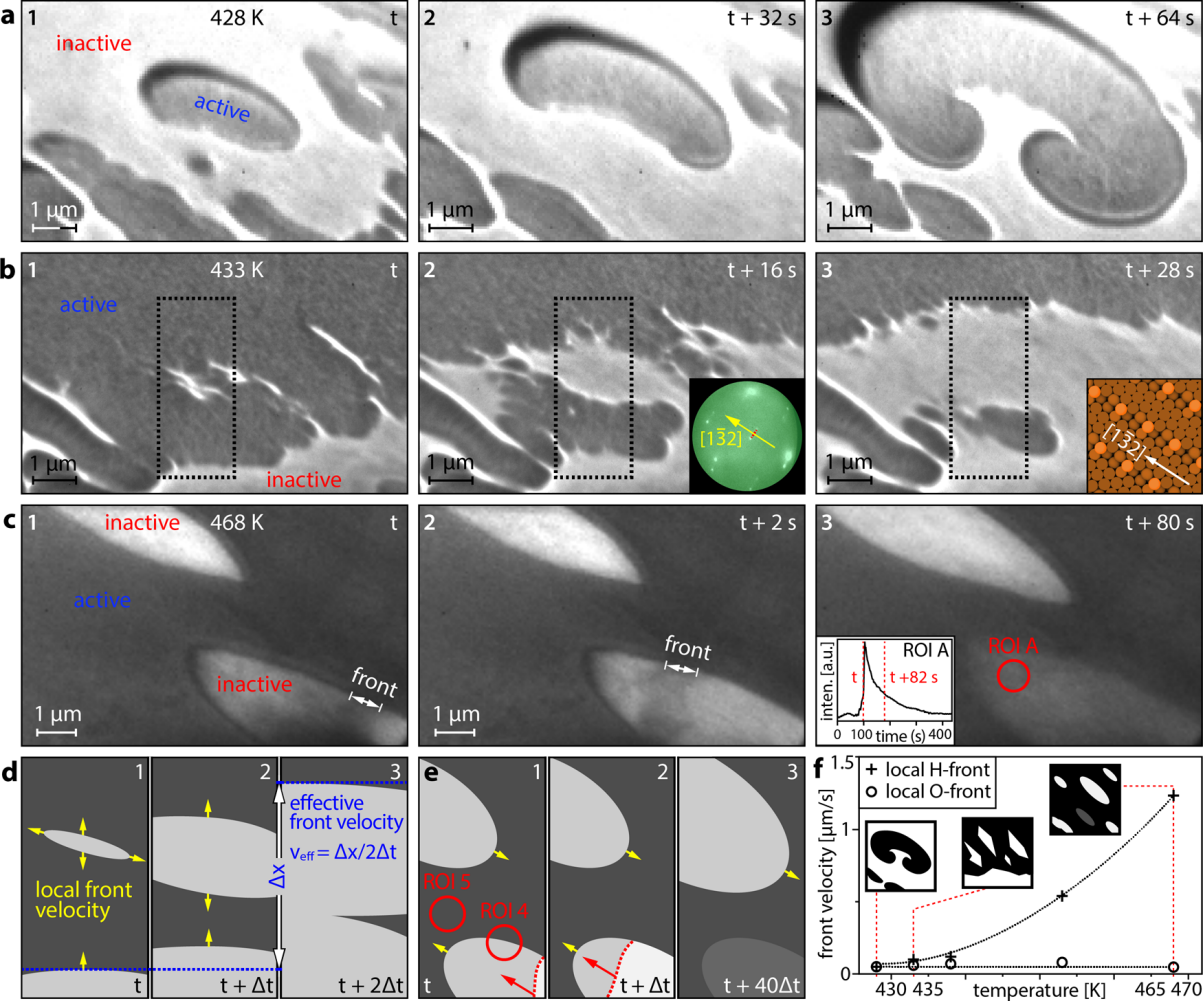
Pattern formation in catalytic H_2_ oxidation on Rh(7 5 4) visualized by LEEM at constant *p*H_2_ = 4.5 × 10^–7^ mbar and *p*O_2_ = 5.0 × 10^–7^ mbar: (a) rotating double spiral formed at 428 K. Catalytically inactive regions appear bright, while active regions appear dark in the LEEM video frames (electron energy 2.5 eV); (b) island-assisted front propagation at 433 K. The inset in frame 2 shows a μ-LEED image taken from the same region at *E* = 330 eV, the red dotted line illustrates the spot splitting, and the yellow arrow shows the step direction. The inset in frame 3 shows the corresponding surface structure; (c) collapse of an oxygen island before it can coalesce with other islands at 468 K. The position of the hydrogen front is indicated in frames 1 and 2. The inset in frame 3 shows the intensity recorded in ROI A during the kinetic transition, the time points corresponding to frames 1 and 3 are indicated, all frames in panel (c) are recorded at an electron energy of 3.3 eV; (d) schematic illustration of the island-assisted front propagation, each third corresponds to the black dotted rectangles in frames 1–3 in panel (b); (e) schematic illustration of the oxygen island collapse, ROIs 4 and 5 schematically represent the corresponding ROIs in Figure 2b; (f) local velocities of the hydrogen (crosses) and oxygen (circles) fronts. The cartoon insets depict the different patterns shown in panels (a) to (c). The red dotted lines indicate the temperatures at which the sketched patterns are observed, and the black dotted lines serve as guides for the eye.

Understanding the image contrast in LEEM allows spatially resolved studies of local instabilities with ∼20 nm resolution (at the given magnification), which were merely time-resolved in PEEM (Figure 2e). Figure 4 shows the formation of spatio-temporal patterns at different temperatures in the range 428–468 K, with the reactant pressures kept constant at *p*H_2_ = 4.5 × 10^–7^ mbar and *p*O_2_ = 5.0 × 10^–7^ mbar. As an example, Figure 4a displays how during a kinetic transition at 428 K accompanying each oscillation cycle, the propagating hydrogen and oxygen fronts form a rotating double spiral pattern, a phenomenon known from PEEM studies to occur on a much larger length scale.^48^

Figure 4b illustrates the propagation of the oxygen front at 433 K, which proceeds in an unusual way: ahead of the front, due to the anisotropy of the Rh(7 5 4) surface, plenty of elongated oxygen islands nucleate and grow (frame 1 in Figure 4b), forming oxygen archipelagos ahead of the traveling front (frame 2). Eventually, the archipelagos conglomerate via the development of dendritic structures and merge when the moving oxygen front reaches the oxygen islands (frame 3). To our knowledge, such an island-assisted propagation mechanism was not yet observed, neither in H_2_ oxidation nor in any other heterogeneous catalytic reaction. Each nucleating island possesses its “local front” along the island perimeter, propagating with its local velocity v_loc_, which is different from the effective velocity v_eff_ of the mesoscopically observed front (without resolving the nucleating islands). Figure 4d shows a schematic drawing of this concept, where v_eff_ = 3·v_loc_ by the formation of a singular island ahead of the moving front. For the hydrogen front, such island-assisted front propagation was not observed; therefore, v_eff_ is equal to v_loc_ for hydrogen.

The unique ability of the SMART microscope to directly correlate the orientation of real space images (LEEM) with diffraction patterns obtained for the reciprocal space (μ-LEED)^42,43^ allows us to identify the preferential direction of the front propagation. The latter appears to be along the [1 1 1]-type terraces of the stepped Rh(7 5 4) surface, i.e., in the [1 3̅ 2]-direction, as illustrated by the insets in Figure 4b (details of the crystallographic analysis and μ-LEED patterns of the studied surface regions are given in the Supporting Information). Such preferential front propagation along the terraces of stepped Rh surfaces was previously observed in H_2_ oxidation and is caused by anisotropy of the diffusional hydrogen supply along vs across the atomic steps.^19^

At an even higher temperature of 468 K, kinetic transitions to the active state occur via fast “island-snapping” hydrogen fronts (Figure 4c and schematic illustration in Figure 4e) without island agglomeration. In video frames 1 and 2 of Figure 4c, the fast front appears as a stripe and not as a line due to the blurring caused by the exposure time of 2 s for each LEEM video frame. The origin of such island-mediated transition lies in the subsurface oxygen formation/depletion, which serves as a feedback and “clock” mechanism governing the frequency of the self-sustained oscillations in H_2_ oxidation on Rh.^13−16^ At rising temperatures, the formation/depletion of subsurface oxygen occurs faster and the clock frequency increases, eventually leading to kinetic transitions within the growing oxygen islands before they can coalesce with other islands. The LEEM videos of the three different types of pattern formation at 428, 433, and 468 K are given in the Supporting Information. Such transitions can be registered even in PEEM as sawtooth-like local oscillations (ROI 4 in Figure 2e).

The sawtooth shape of the intensity curves, shown in the inset of frame 3 in Figure 4c, reflects the fast “avalanche-like” snapping of the oxygen islands by hydrogen fronts and the following relatively slow relaxation due to subsurface oxygen depletion, as schematically shown in Figure 4e. The temperature dependence of the local front velocities for oxygen and hydrogen is displayed in Figure 4f. While the local hydrogen front velocity shows an exponential increase with rising temperature, the local velocity of the oxygen front remains approximately constant. The temperature-dependent differences in the local velocities of reaction fronts during self-sustained oscillations allow us to explain the formation of qualitatively different patterns at different temperatures. At 428 K, the local velocities of hydrogen and oxygen fronts are equal, which favors the formation of spiral patterns,^48^ as observed in the present experiments (Figure 4a). At 433 K, the local hydrogen front is about two times faster than the local oxygen front, which can only partially be compensated by island-assisted propagation; thus, no spiral nuclei are formed anymore,^49^ favoring the island-assisted oxygen front propagation in the form of a “frayed” inactive area (frame 3 in Figure 4b). At 468 K, the discrepancy of local velocities appears to be too high for the formation of a mesoscopic front: the growing oxygen islands are “snapped” by fast island-internal hydrogen fronts before they can merge. Therefore, mesoscopic fronts are not visible in PEEM within ROI 3 in Figure 2 despite plenty of local instabilities occurring on a smaller length scale at the present conditions. Since the nucleation of oxygen islands occurs most probably via reaction-induced fluctuations,^18^ the nucleating/collapsing islands are stochastically distributed over the surface, occasionally leaving space for regions that also locally remain temporarily nonoscillating, as visible in ROI 5 in Figure 2e.

### Modeling

To rationalize the experimental findings and to investigate the influence of differences between the front propagation of hydrogen and oxygen on the observed pattern formation, theoretical simulations were performed. Usually, a mean-field approach, which ignores the nonideality of surface rate processes and the stochastic components of adsorption, desorption, and reaction, is used for microkinetic modeling of pattern formation. The alternative use of the Monte Carlo method, which is superior for the simulation of pattern formation on the nm scale,^11^ is still a challenge for simulation of patterns on the μm scale because of limitations in lattice size, rate constants, and calculations of the elementary steps of diffusion. Therefore, a novel model was developed based on a hybrid approach of complementary microkinetic modeling (MKM) and Monte Carlo (MC) simulations, with the goal of realistically describing the experimental microscopy observations. The Langmuir–Hinshelwood mechanism that is well established for catalytic hydrogen oxidation and the formation and depletion of subsurface oxygen as feedback mechanism of the oscillations were assumed. The calculations provide the time-dependent spatial distribution of the catalytically active and inactive states and, thus, allow monitoring the simulated pattern formation, in a kind of virtual reaction microscopy.

In the MC calculations, a hexagonal tiling MC-grid consisting of 160 000 tiles corresponding to a 40 × 34.6 μm^2^ area of the sample surface was used. A simplified MC approach was chosen, where the state of each of the tiles is either catalytically active (low O and H coverage) or catalytically inactive (high O coverage). Transitions between these states occur via two possible pathways: internally, by local oxygen or hydrogen adsorption, which is mediated by the local O_sub_ concentration, or externally, via reaction/diffusion fronts propagating from neighboring grid points. These events are governed by MC, while MKM is used to determine the local probabilities for each event to occur and to calculate the formation/depletion of O_sub_ based on the current local coverages (details of the calculations are given in the Supporting Information).

**Figure 5:**
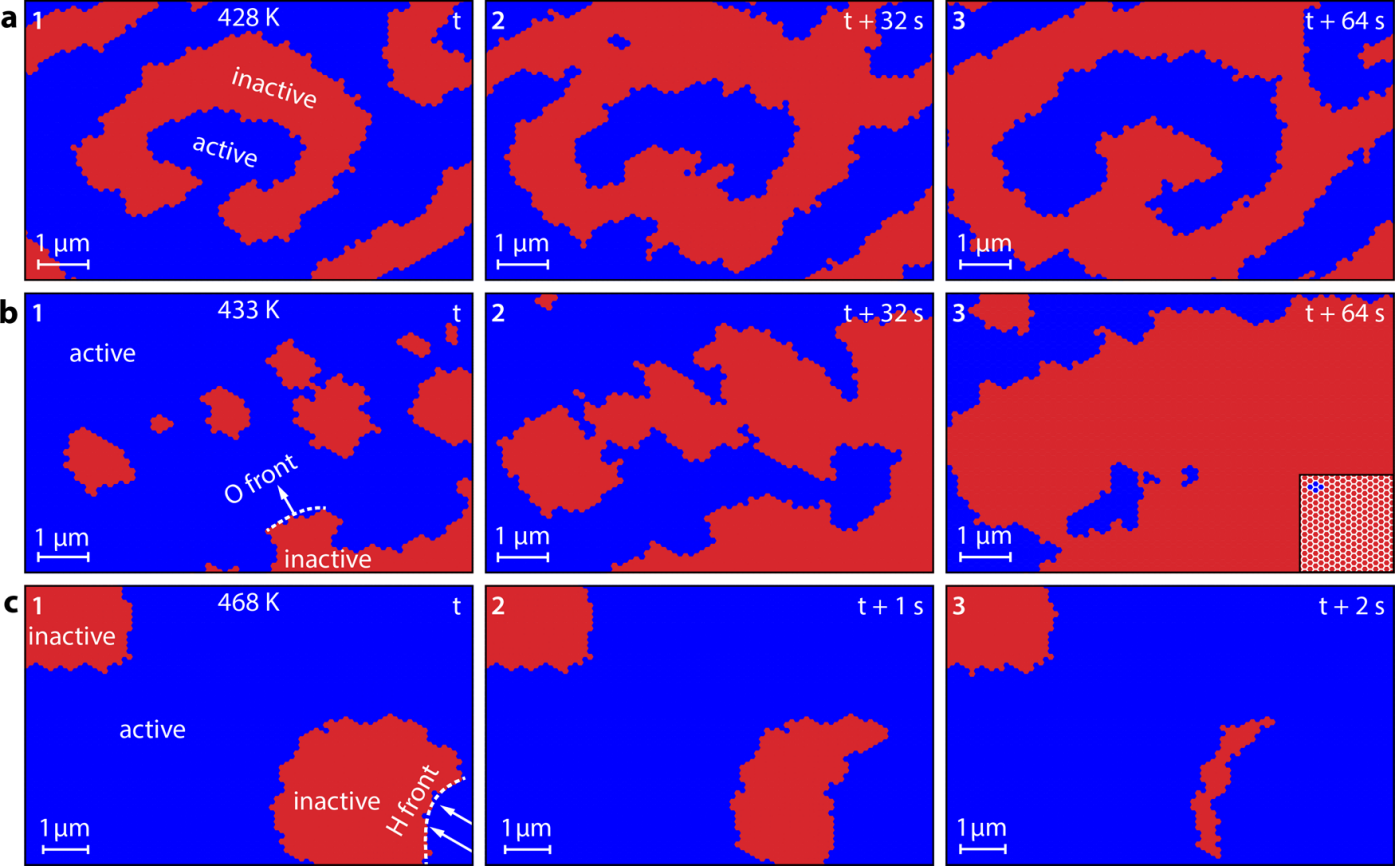
Virtual reaction microscopy of catalytic H_2_ oxidation on Rh at constant *p*H_2_ = 4.5 × 10^–7^ mbar and *p*O_2_ = 5.0 × 10^–7^ mbar: (a) calculated pattern formation at 428 K. Catalytically active regions (low hydrogen and oxygen coverage) are colored blue, and catalytically inactive regions (oxygen-covered) are colored red. A double spiral is recognizably formed in the middle of the image; (b) island-assisted front propagation at 433 K: the white dotted line marks the main front position, the inset in frame 3 shows a part of the hexagonal tiling MC-grid; (c) simulation results at 468 K: the oxygen islands are snapped by hydrogen fronts (white dotted line) before the islands can grow together.

Simulations were performed for the same partial pressures of reactants as used in the experiment (*p*H_2_ = 4.5 × 10^–7^ mbar and *p*O_2_ = 5.0 × 10^–7^ mbar), only varying the temperature. For all calculations, the same set of MKM parameters was used (details are given in the Supporting Information). Results of the simulations are displayed in Figure 5. Figure 5a shows the formation of patterns at *T* = 428 K, where characteristic double spirals are formed. Both hydrogen and oxygen fronts propagate with the same velocity. The simulations closely mirror the pattern formation observed in the experiment at the same temperature (Figure 4a).

At 433 K, hydrogen fronts propagate much faster, but oxygen fronts keep their velocity which is now about one-third of that of hydrogen. The simulations predict the formation and growth of additional small oxygen islands ahead of the main front in the right lower corner of frame 1 (Figure 5b). Eventually, the main oxygen front fuses with these islands (frames 2 and 3). Incorporation of islands contributes to the oxygen front propagation, thus increasing the effective front velocity, which therefore approaches the hydrogen front velocity. Indeed, such a front propagation mechanism was exactly observed in the experiments as island-assisted front propagation (Figure 4b,d).

When increasing the temperature parameter to 468 K, where the hydrogen front is about 2 orders of magnitude faster than the oxygen front, the calculations provide a very different behavior (Figure 5c). Oxygen islands slowly grow until, eventually, one nucleation spot, preferentially at the edge of the island, spontaneously undergoes a sudden transition to the active state, forming a fast hydrogen front that “devours” the whole oxygen island, preventing the fusion with other islands. Again, the calculations reproduce the specific experimentally observed behavior at the same conditions well (Figure 4c,e).

Since the size of the MC-grid and the dimensions of each grid tile can be chosen arbitrarily, both the size of the field of view and the time interval between calculated images are not limited. This allows calculating a stack of surface snapshots (exemplary presented in Figure 5), which can be assembled to a virtual video file (calculated videos are given in the Supporting Information). By convoluting the adsorbate concentrations with a proper contrast mechanism, different imaging modes can be simulated without experimental limitations, such as the size of the field of view or the duration of the experiment. Additionally, such calculated virtual reaction microscopy videos can be processed using the same tools, which are applied to experimental video data complementing the insights into the observed phenomena.^17,37,50^

## Conclusions

In conclusion, we have performed a microscopic study of pattern formation in H_2_ oxidation on Rh using PEEM, LEEM, and XPEEM in a correlative microscopy approach, taking up important challenges in studying this catalytic surface reaction. Due to their significantly higher lateral resolution, LEEM and XPEEM allowed zooming in on processes observed by PEEM, enabling the detection of an unusual island-mediated oxygen front propagation during kinetic transitions. To rationalize the experimental findings, theoretical simulations were performed using a novel model based on a hybrid approach of microkinetic modeling and Monte Carlo, which allows us to realistically simulate the spatio-temporal surface processes in a kind of virtual reaction microscopy on realistic length and time scales. The results of the calculations agree well with the experimental observations and provide novel insights into the mechanism of pattern formation in catalytic hydrogen oxidation on platinum group metals.

## Supporting Information Available

Details on experimental procedures, determination of the local crystallography by μ-LEED, and model calculations (PDF)
Movie S1: In situ low-energy electron microscopy video at 428 K, showing an evolving double spiral pattern during the self-sustaining oscillations in hydrogen oxidation on a Rh(7 5 4) surface (MP4)
Movie S2: In situ low-energy electron microscopy video at 433 K, showing the island-assisted front propagation mechanism during the self-sustaining oscillations in hydrogen oxidation on a Rh(7 5 4) surface (MP4)
Movie S3: In situ low-energy electron microscopy video at 468 K, showing oxygen “island-snapping” by fast hydrogen fronts during the self-sustaining oscillations in hydrogen oxidation on a Rh(7 5 4) surface (MP4)
Movie S4: Model simulation as a virtual reaction microscopy video at 428 K, showing an evolving double spiral pattern during calculated oscillations of the simulated catalytic activity on a grid of 160 000 individual grid tiles (MP4)
Movie S5: Model simulation as a virtual reaction microscopy video at 433 K, showing the island-assisted front propagation mechanism during calculated oscillations of the simulated catalytic activity on a grid of 160 000 individual grid tiles (MP4)
Movie S6: Model simulation as a virtual reaction microscopy video at 468 K, showing oxygen “island-snapping” by fast hydrogen fronts during calculated oscillations of the simulated catalytic activity on a grid of 160 000 individual grid tiles (MP4)
