# Supplementary material for: Pattern Formation in Catalytic H2 Oxidation on Rh: Zooming in by Correlative Microscopy
Source: ACS Catal. 2022 Sep 19;12(19):11974–83. doi: 10.1021/acscatal.2c03692 (PMC9552168; doi:10.1021/acscatal.2c03692)
Supplement: Supplementary file 1 — cs2c03692_si_001.pdf [file cs2c03692_si_001.pdf]

Supporting Information

Pattern formation in catalytic H_2 oxidation on Rh: Zooming in by correlative microscopy

*Johannes Zeininger[1, ‡], Philipp Winkler[1, ‡], Maximilian Raab[1], Yuri Suchorski[1], Mauricio J. Prieto[2], Liviu C. Tănase[2], Lucas de Souza Caldas[2], Aarti Tiwari[2], Thomas Schmidt[2], Michael Stöger-Pollach[3], Andreas Steiger-Thirsfeld[3], Beatriz Roldan Cuenya[2], and Günther Rupprechter[1, *]*

[1] Institute of Materials Chemistry, TU Wien, Getreidemarkt 9, 1060 Vienna, Austria
[2] Department of Interface Science, Fritz-Haber-Institut der Max-Planck Gesellschaft, Faradayweg 4-6, D-14195 Berlin, Germany
[3] University Service Center for Transmission Electron Microscopy, TU Wien, Wiedner Hauptstraße 8-10, 1040 Vienna, Austria

[*] E-Mail: guenther.rupprechter@tuwien.ac.at
[‡] These authors contributed equally to this work.

Experimental Procedures

Sample Preparation

A polished polycrystalline Rh foil (10 × 12 mm^2, 0.2 mm thickness, 99.99% purity, MaTecK) consisting of crystallographically different µm-sized Rh(hkl) domains was used as sample. It was cleaned in UHV by repeated cycles of Ar^+ ion sputtering (ion energy of 1 keV at 300 K), annealing to 1073–1173 K and consecutive chemical treatment in oxygen ($pO_2 = 5.0 \times 10^{-7}$ mbar at 773 K) and hydrogen ($pH_2 = 5.0 \times 10^{-6}$ mbar at 773 K). Cleanliness of the sample was verified before and after each experiment by X-ray photoelectron spectroscopy (XPS), with post-experimental analysis not indicating any changes in surface composition. The sample temperature was measured by a thermocouple via direct physical contact to the front of the sample (Type K in the PEEM experiments, Type C in the LEEM and XPEEM experiments) and regulated by a PID controller within a range of typically 0.25 K. Characterization of the local sample crystallography was performed by electron backscatter diffraction (EBSD) and spatially resolved low energy electron diffraction (µ-LEED), further details are given below.

PEEM

The PEEM experiments were conducted in a multipurpose UHV setup containing a PEEM and an XPS chamber, interconnected by a sample transfer tunnel. The PEEM chamber is equipped with sample cleaning facilities, a PEEM (Staib Instruments PEEM 150), a deuterium discharge UV lamp (Heraeus D200F, photon energy ~6.5 eV), a quadrupole mass spectrometer (MKS e-Vision 2) and a high purity gas supply system (Ar: 99.9999%, H_2: 99.9995%, O_2: 99.9999%). The XPS chamber is equipped with a hemispherical energy analyzer (Specs Phoibos 100), a twin anode X-ray source (Specs XR-50) and sample cleaning facilities and a high purity gas supply system analogous to those in the PEEM chamber. The ongoing H_2 oxidation reaction, visualized in situ by PEEM, was video recorded by a high-speed CCD camera (Hamamatsu C11440-42U30). The PEEM magnification was calibrated by comparison of PEEM images with optical micrographs of individual domains of the polycrystalline Rh sample. The XPS spectra were acquired from circular areas of 100 µm in diameter, using Mg Kα X-ray radiation, with the energy analyzer oriented perpendicular to the sample surface (take-off angle 0°).

LEEM/XPEEM

The LEEM/XPEEM experiments were carried out using the SMART microscope operating at the UE49-PGM beam line of the synchrotron light source BESSY-II of the Helmholtz Centre Berlin (HZB). The aberration corrected and energy filtered instrument, which has been described in detail elsewhere *(S1, S2)*, combines microscopy (LEEM/XPEEM), low-energy electron diffraction (µ-LEED), and laterally resolved X-ray spectroscopy (µ-XPS). The SMART microscope achieves a maximal lateral resolution of 2.6 nm and 18 nm in LEEM and XPEEM mode, respectively. The system is equipped with gas dosing (Ar, H_2, O_2; purity 99.999%) and sample cleaning facilities.

In LEEM, the low-energy electrons elastically backscattered from the sample surface are exploited for imaging, whereby the bright-field imaging mode with a contrast aperture selecting the specularly reflected electron beam was used. In XPEEM, the sample surface is illuminated

with X-rays and photoemitted electrons are used for imaging. The imaging energy analyzer allows the selection of electrons having a binding energy within a window and using them to form the XPEEM image. The choice of energy windows (Figs. 3d, e of the main text) corresponding to the XPS peaks specific for particular elements and their chemical states provides the chemical contrast (Fig. 3b of the main text).

EBSD

Electron backscatter diffraction (EBSD) is an additional characterization technique based on scanning electron microscopy (SEM), enabling crystallographic microstructural characterization of polycrystalline materials, including determination of individual grain orientations *(S3–S5)*. The backscattered electrons of an electron beam, focused onto a particular sample spot, form a diffraction pattern consisting of Kikuchi bands, which correspond to each of the diffracting crystal lattice planes. Computer-based pattern transformation allows determination of the crystallographic surface orientation of the studied particular sample spot. In the present study, the EBSD characterization was performed by a FEI Quanta 200F field emission scanning electron microscope using standard EBSD conditions and evaluation procedures *(S6)*. A comparison of pre- and post-reaction EBSD characterization has shown that the surface structures of the Rh(hkl) domains remain conserved even after extended periods of the ongoing hydrogen oxidation reaction.

Determination of the local crystallography by μ-LEED

The local crystallographic orientation of a specific domain region of the polycrystalline Rh sample investigated in the LEEM studies was determined using μ-LEED. The determination is based on the fact that any regular atomic stepped array found on a stepped surface produces a characteristic LEED pattern *(S7, S8)*. For the μ-LEED, first, a specific area of interest with 1.5 μm in diameter was selected by LEEM. From this area, LEED patterns were obtained for different electron energies in the range of 20 to 350 eV. Figure S1a exemplarily displays the LEED pattern obtained at 330 eV, showing a six-fold symmetry which reflects the [1 1 1]-like nature of the surface. A closer look reveals the splitting of LEED spots, indicating a stepped surface. Such a stepped surface can be considered as consisting of two superimposed lattices: the short-period lattice associated with the atomic structure of the terraces and the long-period lattice (vicinal plane) associated with the atomic steps. In the corresponding Ewald construction, both of these lattices contribute with reciprocal rods which are tilted by the angle between the vicinal planes and the terraces and have a certain thickness due to the non-negligible terrace width *(S9)*. This is reflected in the LEED pattern by a characteristic spot splitting (Fig. S1a) that depends on the primary electron energy. The inset in Fig. S1a shows the extension of the spot splitting line (red dotted line) in the $[5\ \bar{1}\ \bar{4}]$-direction (towards the $[4\ 0\ \bar{2}]$ spot). The width, the direction and the energy dependence of the splitting allow determination of the crystallographic orientation of the selected area.

Figure S1b shows the application of this concept to the present μ-LEED data: first, the experimentally obtained spot existence regions (marked by white bars in the inset of Fig. S1b) were summarized in a diagram. Then, the Ewald geometric construction was composed, where

the tilted black lines result from the atomic step-edges and the blue bars result from the terraces which, as mentioned above, have an extended width in the real space, in contrary to step-edges. The resulting construction provides the [14 10 8]-direction as the best fit for the spot existence diagram in the inset of Fig.1Sb, which corresponds to a (7 5 4) surface orientation for the studied μm-sized surface region. Fig. S1c displays a corresponding ball model of the Rh(7 5 4) surface. Notably, the SMART instrument uses an electrostatic lens system without image rotation well-known for systems with magnetic lenses, i.e. the step orientation obtained from the μ-LEED can be unambiguously associated with the LEEM images. In order to ensure the homogeneity of the studied surfaces, μ-LEED patterns were recorded for several μm-sized surface areas placed at distances of a few μm in the region displayed in Figs. 3 and 4 (Fig. S1d-f).

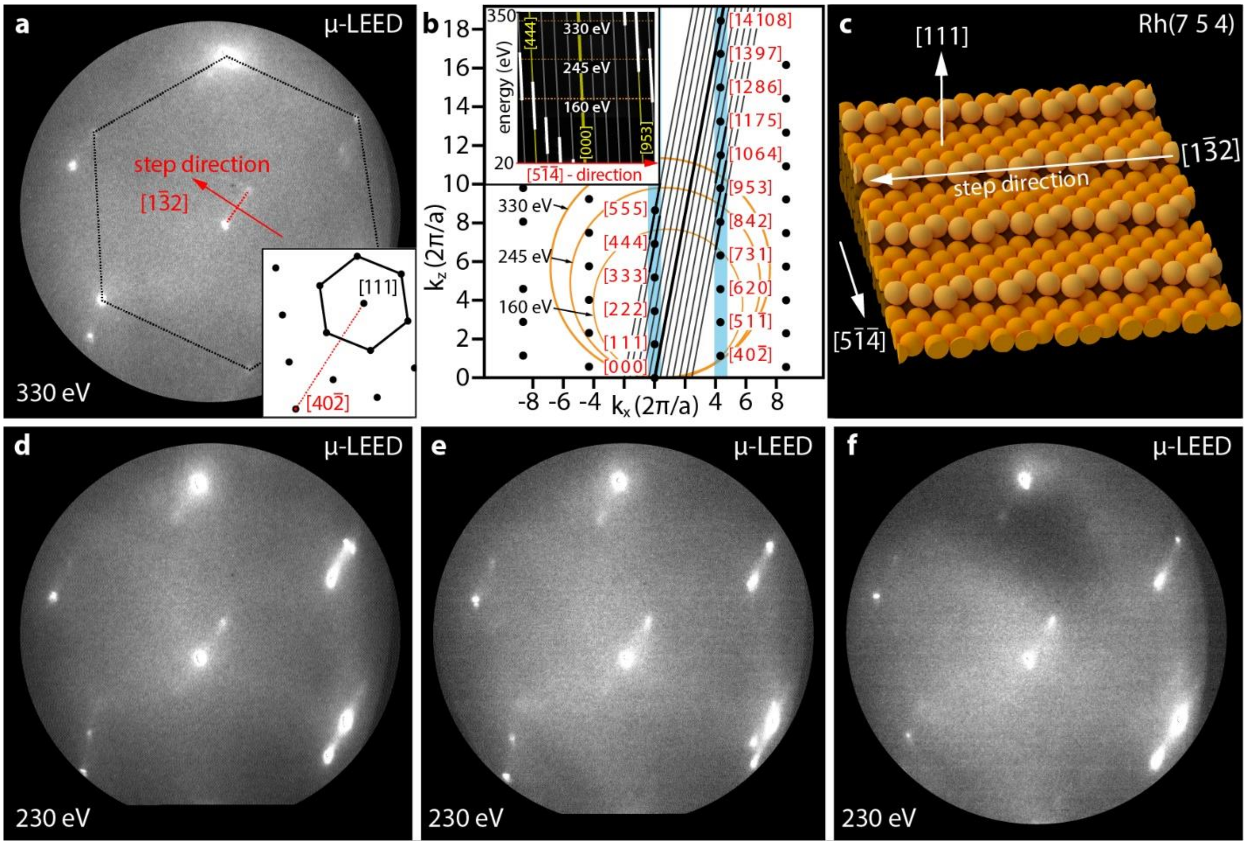

Figure S1. Determination of the local crystallographic orientation by μ-LEED: (a) hexagonal μ-LEED pattern obtained from the selected region on the Rh domain studied by LEEM (electron energy 330 eV). Spot splitting is well visible (exemplarily marked by the red dotted line), the inset schematically shows the spot splitting direction; (b) geometric Ewald construction of electron diffraction. Projections of Ewald spheres for 160, 245 and 330 eV are shown as orange lines. The inset shows the spot existence diagram in the energy range from 20 to 350 eV. The horizontal orange dotted lines correspond to the electron energies, for which the Ewald spheres are drawn; (c) ball model of the Rh(7 5 4) surface with the step direction indicated; (d) the same as in (a) but with an electron energy of 230 eV; (e) the same as in (d) but for a μm-sized region located 5 μm to the right of (d); (f) the same as in (d) but for a μm-sized region located 5 μm below (e).

Model simulations

The modeling approach used for the present simulations of pattern formation during the self-sustaining oscillations in hydrogen oxidation on rhodium is based on the Langmuir-Hinshelwood mechanism that is well established for this catalytic reaction *(S10)*. The local formation and depletion of subsurface oxygen acts as feedback mechanism, governing the transitions from the inactive to the active state of the local catalytic activity and vice versa *(S11–S15)*. The oscillation cycle can be described as follows: Presence of molecular hydrogen and oxygen in the gas phase results in their competitive coadsorption on rhodium surface. As the initial sticking coefficient of oxygen is much higher than that of hydrogen, at the experimental partial pressures $pH_2 = 4.5 \times 10^{-7}$ mbar and $pO_2 = 5.0 \times 10^{-7}$ mbar, oxygen preferentially adsorbs to the surface via a molecular precursor, which dissociates into two chemisorbed oxygen atoms. Since hydrogen adsorption is generally blocked by adsorbed oxygen at these conditions *(S16)*, this results in a catalytically inactive state (Fig. S2; 1). After a dense oxygen coverage is formed, adsorbed oxygen atoms can migrate under the topmost rhodium layer, forming subsurface oxygen (Fig. S2; 2). The presence of subsurface oxygen reduces the sticking coefficient of oxygen, increasing the probability of hydrogen adsorption. This leads, upon surpassing a critical threshold of subsurface oxygen coverage, to a switch in the competitive coadsorption in favor of hydrogen and thus to a kinetic transition to the catalytically active state. In the active state, both hydrogen and oxygen can adsorb on the surface, where they react and form water which immediately desorb from the surface at the present conditions (Fig. S2; 3). Due to the continuous formation of water, the surface coverage of both hydrogen and oxygen remains low, thus subsurface oxygen can now migrate back to the surface.

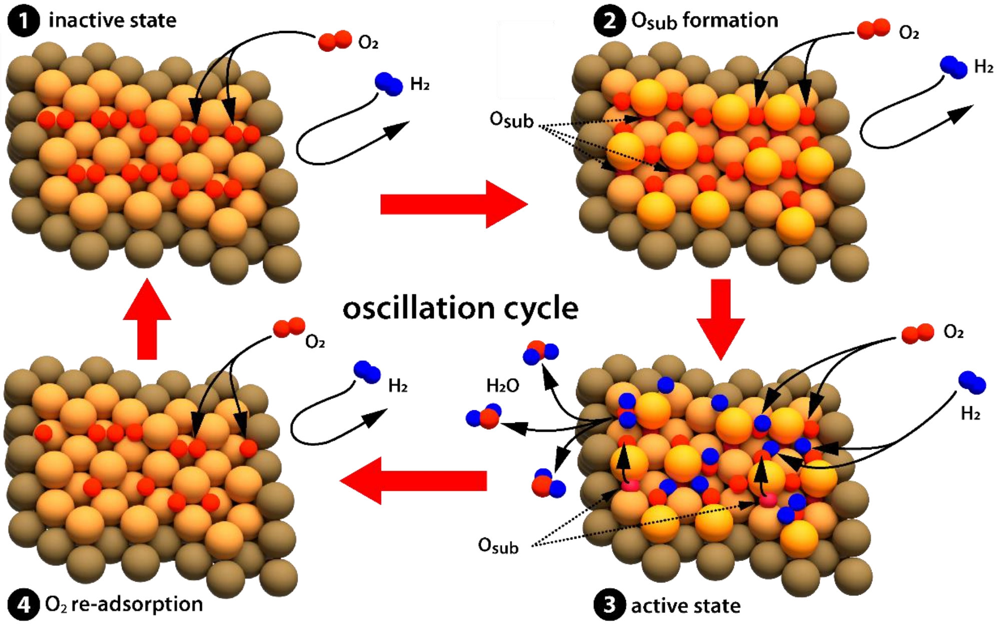

Figure S2: Schematic illustration of the oscillation mechanism for the catalytic hydrogen oxidation on rhodium. Color code: golden – Rh, red – O, blue – H.

The depletion of the subsurface oxygen depot leads to a recovery of the oxygen sticking, thus raising the favorability of oxygen adsorption (Fig. S2; 4). Eventually, oxygen again adsorbs preferentially and covers the surface, returning the system to its initial inactive state and closing the oscillation cycle.

The key role in the above oscillating process is played by the formation/depletion of subsurface oxygen: surpassing a high critical amount of subsurface oxygen causes a transition to the active catalytic state, while a fall below a certain low subsurface oxygen coverage induces a transition to the inactive state. The rate of the subsurface oxygen formation/depletion, which depends on the surface structure, governs, as a feedback mechanism, the frequency of the oscillations.

Based on this view of catalytic hydrogen oxidation, the present model was developed as a hybrid between micro-kinetic modeling (MKM) and Monte Carlo simulations (MC). The goal of our approach was to achieve a model description of the observed phenomena which can directly be compared to experiments.

The model approach assumes that kinetic transitions between states of catalytic activity can be narrowed down to two options: internal "self-induced" transitions through adsorption based on the local coverage of subsurface oxygen (θ_S^i) as described above, and externally induced transitions, caused by propagation of reaction/diffusion fronts from adjacent surface sites. Both of these transitions can be described by a MC approach, with the probabilities for each local transition event to occur in a MC step based on the local amount of θ_S^i. Both the local coverage of subsurface oxygen and the local transition probabilities are updated before each MC step by MKM calculations, with a time interval (Δt) of 40 ms between each MC step used for the calculations.

As a grid for the Monte Carlo simulation, a 400 x 400 hexagonal tiling grid was chosen, representing the (1 1 1) fcc surface structure of the terraces forming the Rh(7 5 4) surface. Due to the close-packing of the 160000 grid tiles, this results in a rectangular model field of view. To realistically simulate the experimental LEEM observations, the size of this field of view was set to 40 x 34.6 μm^2, so each of the grid tiles contains a surface area of about 8660 nm^2, or roughly 86600 surface sites, under the assumption of a surface site area of 0.10 nm^2 *(S10)*. Based on the above-mentioned view of the mechanism of the reaction, it is reasonable to assign each grid tile a state of catalytic activity, active or inactive, each represented by a distinct set of surface coverages (inactive – high oxygen coverage, few empty adsorption sites; active – low oxygen and hydrogen coverage, many empty adsorption sites, see Tab. S3).

The local formation and depletion of subsurface oxygen can be accurately described via micro-kinetic modelling, as has been previously applied *(S10–S15, S17)*. This deterministic approach is also suitable to describe the local subsurface oxygen coverage of each grid tile. The rate of the subsurface oxygen formation and depletion at the present conditions is sufficiently high to be accurately calculated by a numerical integration of the MKM differential equations, even disregarding the stochastic part of the local subsurface oxygen formation/depletion on each surface site of the grid tile.

In our model, the subsurface oxygen coverage θ_S^i of each grid tile i is described by the following kinetic equation:

$$\frac{d\theta_S^i}{dt} = k_{ox}\theta_O^i(1-\theta_S^i) - k_{red}^i\theta_S^i\theta_*^i\,, \qquad \text{(Eq. S1)}$$

where θ_O^i is the local oxygen coverage and θ_*^i represents the coverage of empty surface sites. The rate and coupling constants are given by

$$k_{ox} = k_{ox}^0 e^{-\beta E_{ox}^i}, \qquad \text{(Eq. S2)}$$

$$k_{red}^i = k_{red}^0 e^{-\beta\left(E_{red}^i + A_{red}^s\theta_S^i\right)}, \qquad \text{(Eq. S3)}$$

where $\beta = 1/k_\mathrm{B}T$. The values of the kinetic parameters used in the present calculations are given in Tab. S3 and are based on the work of McEwen *et al. (S10, S17)*. The rate of subsurface oxygen formation/depletion depends on the local activation energy for the formation of subsurface oxygen (E_{ox}^i in the model equations) and thus on the crystallographic orientation of the surface *(S11–S15)*. Each grid tile is originally assigned the E_{ox}^i value of the crystallographically regular surface, with local deviations due to local defects of the crystallographic surface structure, randomly distributed across the surface. The occurrence rate of such modified E_{ox}^i values, as well as the E_{ox}^i value of the regular surface, are given in Tab. S1. For all calculations, the same distribution of E_{ox}^i values was used.

Table S1. Surface distribution probabilities of the local activation energy E_{ox}^i for the formation of subsurface oxygen.

**occurrence rate**	$\boldsymbol{E_{ox}^i}$ **(eV)**
regular surface	1.25
6.3 x 10^{-6}	1.15
5.3 x 10^{-2}	1.172
2.5 x 10^{-3}	1.30

The local activation energy for the depletion of subsurface oxygen E_{red}^i was calculated from the respective E_{ox}^i, utilizing the linear relation from Suchorski *et al. (S12)*

$$E_{red}^i = 0.293 + 0.776\,E_{ox}^i. \qquad \text{(Eq. S4)}$$

The two possibilities for local transition events to occur (internally via the adsorption, mediated by the formation and depletion of subsurface oxygen, or externally via a reaction/diffusion front from neighboring grid tiles) are incorporated in the MC simulation model as follows: Although the adsorption of hydrogen is generally blocked on the oxygen covered surface, there still exists a certain probability of hydrogen adsorption due to a few stochastically appearing empty sites on

the surface. The local internal transition to the active state is governed by the probability of hydrogen adsorption φ_{Hads} on the oxygen covered grid tile. This probability is assumed to be proportional to the rate of hydrogen adsorption that can also be derived from the MKM *(S10, S17)* by

$$\varphi_{Hads,t} = \int_t^{t+\Delta t} 2k_a^H p_{H_2} \theta_*^{i^2}\, dt, \qquad \text{(Eq. S5)}$$

with

$$k_a^H = S_0^H\, a_s / \sqrt{2\pi m_{H_2}/\beta}, \qquad \text{(Eq. S6)}$$

where S_0^H corresponds to the initial sticking coefficient of hydrogen, a_s denotes the area of a surface site and $m_{\mathrm{H}_2}$is the average molecular mass of hydrogen. The probability for a transition to the active state is

$$\chi_{Hads,t} = N \cdot q_H \cdot \varphi_{Hads,t} \cdot \rho_H, \qquad \text{(Eq. S7)}$$

where N is the number of surface sites per grid tile. The adsorption of hydrogen and the following reaction to water leaves empty surface sites. These empty sites can subsequently be occupied by hydrogen, leading, after reaction to water, to even more empty surface sites, and thereby to an autocatalytic reaction and a kinetic transition to the active state. However, the empty surface sites can instead also be refilled by adsorbing oxygen, returning the surface coverage to its initial state. q_H is a parameter describing the probability that hydrogen adsorption on an oxygen covered grid tile leads to a successful kinetic transition. ρ_H is a factor describing the dependence of the probability of a hydrogen caused transition to the active state on the local subsurface oxygen coverage, which is written as

$$\rho_H = ln\left(\frac{A_1 \cdot \theta_S^i}{A_{lim}} - A_1 + 1\right) \Big/ ln\left(-A_1 + 1 + \frac{A_1}{A_{lim}}\right), \qquad \text{(Eq. S8)}$$

where A_{lim} is the subsurface oxygen limit needed for a transition to the active state, and A_1 is a factor describing the exponential dependence of the transition probability on θ_S^i. For a saturated subsurface coverage, ρ_H becomes equal to one.

The same can also be done for the internal transition to the inactive state, caused by the favored adsorption of oxygen. The probability of oxygen adsorption on a surface site of the grid tile is derived from the MKM as

$$\varphi_{Oads,t} = \int_t^{t+\Delta t} \frac{2 \cdot k_a^O K^i p_{O_2} \theta_*^{i^2}}{1 + K^i \theta_*^{i^2}}\, dt, \qquad \text{(Eq. S9)}$$

with

$$k_a^O = S_0^O\, a_s / \sqrt{2\pi m_{O_2}/\beta}, \qquad \text{(Eq. S10)}$$

$$K^i = K_0 e^{-\beta\left(E_K + A_K^O \theta_O^i + A_K^S \theta_S^i\right)}, \qquad \text{(Eq. S11)}$$

where the parameters S_0^{O} and m_{O_2} represent the initial sticking coefficient of oxygen and its molecular mass, respectively. The probability for a transition to the active state is

$$\chi_{Oads,t} = N \cdot q_O \cdot \varphi_{Oads,t} \cdot \rho_O, \qquad \text{(Eq. S12)}$$

where q_O is a parameter describing the probability that the amount of adsorbed oxygen on a grid tile is sufficient for a kinetic transition to the inactive state, despite it being partially consumed in the reaction until the completed kinetic transition. ρ_O is a factor describing the dependence of the transition probability on the local subsurface concentration as

$$\rho_O = ln\left(\frac{-B_1 \cdot \theta_S^i}{B_{lim}} + B_1 + 1\right) / ln(B_1 + 1), \qquad \text{(Eq. S13)}$$

where B_{lim} is the subsurface oxygen limit needed for a transition to the active state, and B_1 is a parameter describing the exponential dependence of the transition probability on θ_S^i. When no subsurface oxygen is present, ρ_O becomes equal to one.

Apart from these adsorption-induced kinetic transitions, externally induced transitions are possible via reaction/diffusion fronts. In the kinetic transition to the active state, the hydrogen front propagation can be considered as propagation from one grid tile to a neighboring tile with a certain front velocity. In the model, this is represented by a local *event of front propagation*, in which a grid tile adopts the state of catalytic activity of its neighbor. For such a transition to occur, it is necessary that at least one neighboring grid tile is in a *front state*, meant as state of a tile immediately after the kinetic transition to the active state. The probability of an event of front propagation which induces a transition for the nearest neighboring grid tile determines the local velocity of the front, which can be extracted from the experimental observations. The corresponding relation between the probability for an inactive grid tile to turn active and the front velocity is then

$$\chi_{H,fr} = v_H \cdot n_{H,fr} \cdot X_{H,fr} \cdot \Delta t, \qquad \text{(Eq. S14)}$$

where v_H is the front velocity parameter of the hydrogen front and $n_{H,fr}$ is the number of neighboring grid tiles being already in an active state front state. $X_{H,fr}$ is a factor based on $n_{H,fr}$, given in Tab. S2.

Analogously, the corresponding relation between the probability for an active grid tile to turn inactive via oxygen front propagation and the front velocity is

$$\chi_{O,fr} = v_O \cdot n_{O,fr} \cdot X_{O,fr} \cdot \Delta t, \qquad \text{(Eq. S15)}$$

where v_O is the front velocity parameter of the oxygen front, $n_{O,fr}$ is the number of neighboring grid tiles in an inactive front state and $X_{O,fr}$ is a factor based on $n_{O,fr}$, also given in Tab. S2.

Table S2. Front propagation factors for the hydrogen and oxygen fronts based on the number of front state neighbors.

n	1	2	3	4	5	6
$X_{H,fr}$	0.9	1.8	3.5	8	14	20
$X_{O,fr}$	1	1.5	2.5	6	16	25

By choosing the size of each grid tile and the grid resolution, as well as the time delay for the numerical integration of the MKM differential equations, the chosen spatial resolution as well as time resolution can be directly assigned to each calculated MC model step, thereby generating time-series of surface images which can be interpreted as series of frames of a *simulated virtual surface microscopy* (see Fig. 5 in the main text and supporting movies 4-6).

Table S3. Micro-kinetic model parameters and Monte Carlo simulation parameters used in the present simulations of the LEEM patterns. Energies are given in eV, rate constants in 1/s and v_H and v_O in grid tiles/s.

symbol	parameter description	T = 428 K	T = 433 K	T = 468 K
k_{ox}^{0}	pre-factor for oxygen diffusion from surface to subsurface sites	1.00 x 10^{12}	1.00 x 10^{12}	1.00 x 10^{12}
k_{red}^{0}	pre-factor for oxygen diffusion from subsurface to surface sites	3.00 x 10^{12}	3.00 x 10^{12}	3.00 x 10^{12}
A_{red}^{s}	subsurface oxygen coverage dependence of the activation energy for subsurface oxygen depletion	-0.0200	-0.0200	-0.0200
S_0^H	initial sticking coefficient of H	0.300	0.300	0.300
q_H	pre-factor for transition to the active state	1.37 x 10^{-4}	1.37 x 10^{-4}	1.37 x 10^{-4}
K_0	pre-factor for oxygen dissociation equilibrium constant	0.2525	0.2525	0.2525
E_k	activation energy for oxygen dissociation equilibrium constant	-0.178	-0.178	-0.178
A_K^O	coverage dependence of adsorbed oxygen on the activation energy of oxygen dissociation	0.158	0.158	0.158
A_K^s	coverage dependence of subsurface oxygen on the activation energy of oxygen dissociation	0.082	0.082	0.082
S_0^O	initial sticking coefficient of O	0.600	0.600	0.600
q_O	pre-factor for transition to the inactive state	2.25 x 10^{-7}	2.25 x 10^{-7}	2.25 x 10^{-7}
θ_O^{act}	surface coverage of O in the active state	0.0500	0.0500	0.0500
θ_*^{act}	empty sites in the active state	0.950	0.950	0.950
θ_O^{inact}	surface coverage of O in the inactive state	0.850	0.830	0.750

Table S3. (continued)

symbol	parameter description	T = 428 K	T = 433 K	T = 468 K
θ_*^{inact}	empty sites in the inactive state	0.150	0.170	0.250
A_1	dependence on θ_S^i for the transition probability to the active state	-3.00	-3.00	-3.00
A_{lim}	subsurface oxygen limit needed for a transition to the active state	0.740	0.735	0.600
B_1	dependence on θ_S^i for the transition probability to the inactive state	10.0	10.0	10.0
B_{lim}	subsurface oxygen limit needed for a transition to the inactive state	0.100	0.100	0.020
v_H	front velocity parameter of the hydrogen front	0.00300	0.00700	0.225
v_O	front velocity parameter of the oxygen front	0.00300	0.00300	0.00300

References

(S*1*) Fink, R.; Weiss, M. R.; Umbach, E.; Preikszas, D.; Rose, H.; Spehr, R.; Hartel, P.; Engel, W.; Degenhardt, R.; Wichtendahl, R.; Kuhlenbeck, H.; Erlebach, W.; Ihmann, K.; Schlögl, R.; Freund, H.-J.; Bradshaw, A. M.; Lilienkamp, G.; Schmidt, Th.; Bauer, E.; Benner, G. SMART: A Planned Ultrahigh-Resolution Spectromicroscope for BESSY II. *J. Electron Spectrosc. Relat. Phenom.* **1997**, *84* (1–3), 231–250. DOI: 10.1016/S0368-2048(97)00016-9.

(S*2*) Wichtendahl, R.; Fink, R.; Kuhlenbeck, H.; Preikszas, D.; Rose, H.; Spehr, R.; Hartel, P.; Engel, W.; Schlögl, R.; Freund, H.-J.; Bradshaw, A. M.; Lilienkamp, G.; Schmidt, Th.; Bauer, E.; Benner, G.; Umbach, E. SMART: An Aberration-Corrected XPEEM/LEEM with Energy Filter. *Surf. Rev. Lett.* **1998**, *05* (06), 1249–1256. DOI: 10.1142/S0218625X98001584.

(S*3*) Weatherup, R. S.; Shahani, A. J.; Wang, Z.-J.; Mingard, K.; Pollard, A. J.; Willinger, M.-G.; Schloegl, R.; Voorhees, P. W.; Hofmann, S. In Situ Graphene Growth Dynamics on Polycrystalline Catalyst Foils. *Nano Lett.* **2016**, *16* (10), 6196–6206. DOI: 10.1021/acs.nanolett.6b02459.

(S*4*) König, U.; Davepon, B. Microstructure of Polycrystalline Ti and Its Microelectrochemical Properties by Means of Electron-Backscattering Diffraction (EBSD). *Electrochimica Acta* **2001**, *47* (1–2), 149–160. DOI: 10.1016/S0013-4686(01)00572-2.

(S*5*) Daviddi, E.; Shkirskiy, V.; Kirkman, P. M.; Robin, M. P.; Bentley, C. L.; Unwin, P. R. Nanoscale Electrochemistry in a Copper/Aqueous/Oil Three-Phase System: Surface Structure–Activity-Corrosion Potential Relationships. *Chem. Sci.* **2021**, *12* (8), 3055–3069. DOI: 10.1039/D0SC06516A.

(S*6*) Humphreys, F. J. Review: Grain and Subgrain Characterisation by Electron Backscatter Diffraction. *J. Mater. Sci.* **2001**, *36* (16), 3833–3854. DOI: 10.1023/A:1017973432592.

(S*7*) Ellis, W. P.; Schwoebel, R. L. LEED from Surface Steps on UO2 Single Crystals. *Surf. Sci.* **1968**, *11* (1), 82–98. DOI: 10.1016/0039-6028(68)90040-X.

(S*8*) Henzler, M. LEED-Investigation of Step Arrays on Cleaved Germanium (111) Surfaces. *Surf. Sci.* **1970**, *19* (1), 159–171. DOI: 10.1016/0039-6028(70)90115-9.

(S*9*) Wagner, F. T.; Ross, P. N. LEED Spot Profile Analysis of the Structure of Electrochemically Treated Pt(100) and Pt(111) Surfaces. *Surf. Sci.* **1985**, *160* (1), 305–330. DOI: 10.1016/0039-6028(85)91044-1.

(S*10*) McEwen, J.-S.; Gaspard, P.; de Bocarmé, T. V.; Kruse, N. Electric Field Induced Oscillations in the Catalytic Water Production on Rhodium: A Theoretical Analysis. *Surf. Sci.* **2010**, *604* (17–18), 1353–1368. DOI: 10.1016/j.susc.2010.04.007.

(S*11*) Suchorski, Y.; Datler, M.; Bespalov, I.; Zeininger, J.; Stöger-Pollach, M.; Bernardi, J.; Grönbeck, H.; Rupprechter, G. Visualizing Catalyst Heterogeneity by a Multifrequential Oscillating Reaction. *Nat. Commun.* **2018**, *9* (1), 600. DOI: 10.1038/s41467-018-03007-3.

(S*12*) Suchorski, Y.; Datler, M.; Bespalov, I.; Zeininger, J.; Stöger-Pollach, M.; Bernardi, J.; Grönbeck, H.; Rupprechter, G. Surface-Structure Libraries: Multifrequential Oscillations in Catalytic Hydrogen Oxidation on Rhodium. *J. Phys. Chem. C* **2019**, *123* (7), 4217–4227. DOI: 10.1021/acs.jpcc.8b11421.

(S*13*) Suchorski, Y.; Zeininger, J.; Buhr, S.; Raab, M.; Stöger-Pollach, M.; Bernardi, J.; Grönbeck, H.; Rupprechter, G. Resolving Multifrequential Oscillations and Nanoscale Interfacet Communication in Single-Particle Catalysis. *Science* **2021**, *372* (6548), 1314–1318. DOI: 10.1126/science.abf8107.

(S*14*) Winkler, P.; Zeininger, J.; Raab, M.; Suchorski, Y.; Steiger-Thirsfeld, A.; Stöger-Pollach, M.; Amati, M.; Gregoratti, L.; Grönbeck, H.; Rupprechter, G. Coexisting Multi-States in Catalytic Hydrogen Oxidation on Rhodium. *Nat. Commun.* **2021**, *12* (1), 6517. DOI: 10.1038/s41467-021-26855-y.

(S*15*) Zeininger, J.; Suchorski, Y.; Raab, M.; Buhr, S.; Grönbeck, H.; Rupprechter, G. Single-Particle Catalysis: Revealing Intraparticle Pacemakers in Catalytic H_2 Oxidation on Rh. *ACS Catal.* **2021**, *11* (15), 10020–10027. DOI: 10.1021/acscatal.1c02384.

(S*16*) Padowitz, D. F.; Sibener, S. J. Kinetics of Hydrogen Oxidation to Water on the Rh(111) Surface Using Multiple Source Modulated Molecular Beam Techniques. *Surf. Sci.* **1991**, *254* (1–3), 125–143. DOI: 10.1016/0039-6028(91)90645-9.

(S*17*) McEwen, J.-S.; Gaspard, P.; Visart de Bocarmé, T.; Kruse, N. Oscillations and Bistability in the Catalytic Formation of Water on Rhodium in High Electric Fields. *J. Phys. Chem. C* **2009**, *113* (39), 17045–17058. DOI: 10.1021/jp901975w.
